# Supplementary material for: Associations between significant head injury in male juveniles in prison in Scotland UK and cognitive function, disability and crime: A cross sectional study
Source: PLoS One. 2023 Jul 12;18(7):e0287312. doi: 10.1371/journal.pone.0287312 (PMC10337871; doi:10.1371/journal.pone.0287312)
Supplement: S1 File — (DOCX) [file pone.0287312.s001.docx]

**S1 Background Supplementary Information**

**Literature Search**

A literature search was carried out to determine whether there is published evidence on disability associated with head injury in juvenile offenders. The following databases were searched from 1 January 1996 to 6 April 2022; EMBASE (OVID), Medline (OVID). Duplicates were removed prior to references being retrieved for review. The text word searches used were as follows:

[brain injur* or head injur*)+[crime or offend*] + disability

[brain injur* or head injur*]+ [juveniles or delinquents]+ disability +prevalence

[brain injur* or head injur*]+ [juveniles or delinquents]+ disability+ [crime or offend*] [brain injur* or head injur*]+ [crime or offend*]+ glasgow outcome scale

No relevant papers were found.

**Analysis of Cognitive Test Scores**

The cognitive test z-scores were adjusted for age, years of education and delayed word memory score This was done by fitting a linear model to the raw cognitive test scores, adjusting for the aforementioned covariates, extracting the residuals and standardising them to mean 0 and SD 1. The overall cognitive impairment z-score was calculated as a mean of the positive z-scores for symbol digit, List Learning, Verbal Fluency (animal and letter) and the negative Trails Part B z-score, resulting in a z-score for which lower values represent greater cognitive impairment. If an individual score was missing, then the overall score was calculated as the mean of the available scores (see table S7 and figures S5-6, pp13-15).

**Assessor Training**

HA and JMcV carried out all participant assessments. HA had carried out assessments on adult male and female prisoners in two previous studies^1^. J McV was a final year clinical psychology trainee. JMcV observed HA assessing a participant and both then carried out and assessment with the other observing and independently scored/rated responses. Concordance was very high. Both were trained/supervised in the use of the assessments by TM. Regular supervision sessions were conducted with TM to discuss any queries in relation to assessment.

^1^ McGinley A, Aslam H, Walker V, McMillan T. Head Injury and Associated Disability in Male Prisoners 0448. *Brain Inj*, 2019; 33,supp1,163-164

McMillan TM, Aslam H, Crowe E, Seddon E, Barry SJE. Associations between significant head injury and persisting disability and violent crime in women in prison in Scotland, UK: a cross-sectional study. *Lancet Psychiatry*; 2021; 8; 512-20

**Methods used to Reduce Error in Self-Report**

| **Variable** | **Tool** | **Method** | **Notes** |
| --- | --- | --- | --- |
| Head Injury | OSU-TBI | Validated interview | Also informing participants about what constitutes a head injury prior to the start of the interview |
| Disability | Glasgow Outcome at Discharge Scale | Validated interview | Also utilising information separately from an informant (Personal Prison Officer) |
