# Supplementary material for: Associations between significant head injury in male juveniles in prison in Scotland UK and cognitive function, disability and crime: A cross sectional study
Source: PLoS One. 2023 Jul 12;18(7):e0287312. doi: 10.1371/journal.pone.0287312 (PMC10337871; doi:10.1371/journal.pone.0287312)
Supplement: S2 File — (DOCX) [file pone.0287312.s002.docx]

**S2 Supplementary Tables**

**Severity of Violent Offences**

**Table S1:** **Most Severe Violent Offence***

|  | **SHI** | **NoSHI** | **Total** |
| --- | --- | --- | --- |
| None | 13 (16%) | 4 (19%) | 17 |
| Less serious assault | 38 (47%) | 7 (33%) | 45 |
| Serious assault | 13 (16%) | 4 (19%) | 17 |
| Severe-endangerment of life | 5 (6%) | 0 (0%) | 5 |
| Murder/ Attempted Murder | 12 (15%) | 6 (29%) | 18 |
|  | 81 | 21 | 102 |

***p= .435**

**Model Fit Statistics for Outcome Measures**

**Table S2: Hosmer-Lemeshow model fit statistics and corresponding p-values for logistic regression models fitted to the outcomes listed, indicating that all models were a good fit to the data**

| **Model** | **Hosmer-Lemeshow statistic** | **p-value** |
| --- | --- | --- |
| **GODS head injury disability**  Current  Historical | 0.66  9.30 | 0.99  0.32 |
| **GODS any cause disability**  Current  Historical | 1.89  1.27 | 0.98  0.99 |
| **Violent offences**  Current  Historical | 2.44  3.88 | 0.96  0.87 |

**Demographics in Scottish Prisons and in the Study Sample**

**Table S3: Comparison of Scottish Prison Statistics (male *arrivals* in Scottish Prisons) for 2019-2020 with the study sample; N (%)**

|  | Scottish prisons | Study sample |
| --- | --- | --- |
| Age band 16-17 | 143 (17) | 19 (18) |
| >17 | 725 (83) | 84 (82) |
| Ethnicity white | 812 (94) | 96 (94) |
| Non-white | 56 (6) | 6 (6) |
| Social Deprivation Quintile |  |  |
| Most deprived- 1 | 388 (45) | 50 (52) |
| 2 | 218 (25) | 23 (24) |
| 3 | 130 (15) | 9 (9) |
| 4 | 56 (6) | 7 (7) |
| Least deprived- 5 | 42 (5) | 7 (7) |
| unknown | 44 (5) | 7 (7) |

Note : The average *daily* attendance was 310

<https://www.gov.scot/publications/scottish-prison-population-statistics/pages/analytical-factors-and-measurements/#Average%20daily%20population>

https://scotland.shinyapps.io/sg-prison-population-statistics/

**Days hospitalised as a result of head injury from self-report**

**Table S4: Estimated time in hospital (days)**

|  | **(N = 98)** | **(N = 77)** | **(N = 21)** |
| --- | --- | --- | --- |
| <1 | 81 (83%) | 61 (79%) | 20 (95%) |
| 1-6 | 13 (13%) | 12 (15%) | 1 (5%) |
| >6 | 4 (4%) | 4 (5%) | 0 (0%) |

**Central Nervous System Disorders and Health**

**Table S5: History of central nervous system (CNS) disorder other than head injury; N (%)**

|  | **All (N = 103)** | **S-HI (N = 82)** | **NoSHI (N = 21)** |
| --- | --- | --- | --- |
| Any CNS diagnosis [excluding HI] | 41 (40%) | 37 (45%) | 4 (19%) |
| ADHD | 29 (28%) | 25 (30%) | 4 (19%) |
| Learning disability | 3 (3%) | 3 (4%) | 0 ( 0%) |
| Autistic Spectrum Disorder | 8 ( 8%) | 7 (9%) | 1 ( 5%) |
| Epilepsy | 4 ( 4%) | 4 ( 5%) | 0 ( 0%) |
| Cerebral anoxia [strangling] | 8 ( 8%) | 7 ( 9%) | 1 ( 5%) |
| Brain infection (meningitis) | 1 ( 1%) | 1 ( 1%) | 0 ( 0%) |
| Stroke or transient ischaemic attack | 0 ( 0%) | 0 ( 0%) | 0 ( 0%) |
| Multiple sclerosis | 0 ( 0%) | 0 ( 0%) | 0 ( 0%) |
| **Toxic hazard**  Household exposure to lead | 0 ( 0%) | 0 ( 0%) | 0 ( 0%) |

**Psychological Trauma**

**Table S6 Traumatic Life Events Questionnaire (TLEQ)**

|  | **Statistic** | **All (N = 103)** | **SHI (N = 82)** | **NoSHI (N = 21)** | **Difference** |
| --- | --- | --- | --- | --- | --- |
| Natural disaster | N_obs_ (N_miss_) | 103 (0) | 82 (0) | 21 (0) |  |
| Yes | N (%) | 3 ( 3%) | 3 ( 4%) | 0 ( 0%) | 4% |
| Motor accident | N_obs_ (N_miss_) | 103 (0) | 82 (0) | 21 (0) |  |
| Yes | N (%) | 32 (31%) | 29 (35%) | 3 (14%) | 21% |
| Other accident | N_obs_ (N_miss_) | 103 (0) | 82 (0) | 21 (0) |  |
| Yes | N (%) | 18 (17%) | 18 (22%) | 0 ( 0%) | 22% |
| Exposure to war | N_obs_ (N_miss_) | 103 (0) | 82 (0) | 21 (0) |  |
| Yes | N (%) | 0 ( 0%) | 0 ( 0%) | 0 ( 0%) | 0% |
| Death of friend/loved one | N_obs_ (N_miss_) | 103 (0) | 82 (0) | 21 (0) |  |
| Yes | N (%) | 89 (86%) | 73 (89%) | 16 (76%) | 13% |
| Loved one accident/assault/illness | N_obs_ (N_miss_) | 103 (0) | 82 (0) | 21 (0) |  |
| Yes | N (%) | 37 (36%) | 32 (39%) | 5 (24%) | 15% |
| Life threatening illness | N_obs_ (N_miss_) | 103 (0) | 82 (0) | 21 (0) |  |
| Yes | N (%) | 6 ( 6%) | 6 ( 7%) | 0 ( 0%) | 7% |
| Robbery with a weapon | N_obs_ (N_miss_) | 103 (0) | 82 (0) | 21 (0) |  |
| Yes | N (%) | 55 (53%) | 47 (57%) | 8 (38%) | 19% |
| Assault by a stranger | N_obs_ (N_miss_) | 103 (0) | 82 (0) | 21 (0) |  |
| Yes | N (%) | 63 (61%) | 52 (63%) | 11 (52%) | 11% |
| Witnessed assault | N_obs_ (N_miss_) | 103 (0) | 82 (0) | 21 (0) |  |
| Yes | N (%) | 76 (74%) | 64 (78%) | 12 (57%) | 21% |
| Threats of serious harm | N_obs_ (N_miss_) | 103 (0) | 82 (0) | 21 (0) |  |
| Yes | N (%) | 76 (74%) | 66 (80%) | 10 (48%) | 32% |
| Physical punishment | N_obs_ (N_miss_) | 103 (0) | 82 (0) | 21 (0) |  |
| No | N (%) | 75 (73%) | 58 (71%) | 17 (81%) |  |
| 1-2 times | N (%) | 2 ( 2%) | 2 ( 2%) | 0 ( 0%) | 2% |
| 3+ times | N (%) | 26 (25%) | 22 (27%) | 4 (19%) | 8% |
| Witnessed family violence | N_obs_ (N_miss_) | 103 (0) | 82 (0) | 21 (0) |  |
| Yes | N (%) | 45 (44%) | 38 (46%) | 7 (33%) | 13% |
| Fear of parental violence | N_obs_ (N_miss_) | 103 (0) | 82 (0) | 21 (0) |  |
| Yes | N (%) | 25 (24%) | 22 (27%) | 3 (14%) | 13% |
| Domestic abuse | N_obs_ (N_miss_) | 103 (0) | 82 (0) | 21 (0) |  |
| No | N (%) | 60 (58%) | 42 (51%) | 18 (86%) |  |
| 1-2 times | N (%) | 20 (19%) | 18 (22%) | 2 (10%) | 12% |
| 3+ times | N (%) | 23 (22%) | 22 (27%) | 1 ( 5%) | 22% |
| Fear of domestic abuse | N_obs_ (N_miss_) | 103 (0) | 82 (0) | 21 (0) |  |
| Yes | N (%) | 8 ( 8%) | 8 (10%) | 0 ( 0%) | 10% |
| Sexual assault as a child | N_obs_ (N_miss_) | 103 (0) | 82 (0) | 21 (0) |  |
| No | N (%) | 95 (92%) | 75 (91%) | 20 (95%) |  |
| 1-2 times | N (%) | 4 ( 4%) | 4 ( 5%) | 0 ( 0%) | 5% |
| 3+ times | N (%) | 4 ( 4%) | 3 ( 4%) | 1 ( 5%) | -1% |
| Sexual assault aged 16 or over | N_obs_ (N_miss_) | 103 (0) | 82 (0) | 21 (0) |  |
| No | N (%) | 95 (92%) | 75 (91%) | 20 (95%) |  |
| 1-2 times | N (%) | 5 ( 5%) | 4 ( 5%) | 1 ( 5%) | 0% |
| 3+ times | N (%) | 3 ( 3%) | 3 ( 4%) | 0 ( 0%) | 4% |
| Fear of sexual abuse | N_obs_ (N_miss_) | 103 (0) | 82 (0) | 21 (0) |  |
| Yes | N (%) | 5 ( 5%) | 4 ( 5%) | 1 ( 5%) | 0% |
| Unwanted sexual attention | N_obs_ (N_miss_) | 103 (0) | 82 (0) | 21 (0) |  |
| Yes | N (%) | 10 (10%) | 8 (10%) | 2 (10%) | 0% |
| Stalking | N_obs_ (N_miss_) | 103 (0) | 82 (0) | 21 (0) |  |
| Yes | N (%) | 18 (17%) | 16 (20%) | 2 (10%) | 10% |
| Miscarriage | N_obs_ (N_miss_) | 103 (0) | 82 (0) | 21 (0) |  |
| Yes | N (%) | 29 (28%) | 25 (30%) | 4 (19%) | 21% |
| Abortion | N_obs_ (N_miss_) | 103 (0) | 82 (0) | 21 (0) |  |
| Yes | N (%) | 10 (10%) | 10 (12%) | 0 ( 0%) | 12% |
| Other | N_obs_ (N_miss_) | 103 (0) | 82 (0) | 21 (0) |  |
| Yes | N (%) | 23 (22%) | 21 (26%) | 2 (10%) | 16% |
|  | | | | |  |

**Table S7: Abuse, trauma and Adverse Childhood Experiences (ACEs)**

| **Variable** | **Statistic** | **All (N = 103)** | **SHI (N = 82)** | **NoSHI (N = 21)** | **Fisher P** |
| --- | --- | --- | --- | --- | --- |
| Any abuse* | N_obs_ (N_miss_) | 103 (0) | 82 (0) | 21 (0) | 0.223 |
| Yes | N (%) | 33 (32%) | 28 (34%) | 5 (24%) | 0.440 |
| TLEQ score | N_obs_ (N_miss_) | 103 (0) | 82 (0) | 21 (0) |  |
|  | Median (IQR) | 6 [4, 9] | 7 [5, 9] | 4 [3, 5] |  |
|  | Range | (1, 15) | (1, 15) | (1, 9) | <0.001 |
| Child abuse** | N_obs_ (N_miss_) | 103 (0) | 82 (0) | 21 (0) |  |
| Yes | N (%) | 33 (32%) | 28 (34%) | 5 (24%) | 0.440 |
| Number of occasions of child abuse | N_obs_ (N_miss_) | 103 (0) | 82 (0) | 21 (0) |  |
| None | N (%) | 70 (68%) | 54 (66%) | 16 (76%) |  |
| One type 1-2 times | N (%) | 4 ( 4%) | 4 ( 5%) | 0 ( 0%) |  |
| One type 3+ times | N (%) | 26 (25%) | 21 (26%) | 5 (24%) |  |
| Both types 1-2 times | N (%) | 0 ( 0%) | 0 ( 0%) | 0 ( 0%) |  |
| Both types, with at least one 3+ times | N (%) | 3 ( 3%) | 3 ( 4%) | 0 ( 0%) | 0.869 |
| Partner violence | N_obs_ (N_miss_) | 103 (0) | 82 (0) | 21 (0) |  |
| Yes | N (%) | 43 (42%) | 40 (49%) | 3 (14%) | 0.006 |
| Number of occasions of partner violence | N_obs_ (N_miss_) | 103 (0) | 82 (0) | 21 (0) |  |
| None | N (%) | 60 (58%) | 42 (51%) | 18 (86%) |  |
| 1-2 times | N (%) | 20 (19%) | 18 (22%) | 2 (10%) |  |
| 3+ times | N (%) | 23 (22%) | 22 (27%) | 1 ( 5%) | 0.015 |
| Total number of ACEs | N_obs_ (N_miss_) | 103 (0) | 82 (0) | 21 (0) |  |
|  | Median (IQR) | 4 [2, 6] | 4 [2, 6] | 3 [1, 6] |  |
|  | Range | (0, 10) | (0, 10) | (0, 8) | 0.195 |
| 4 or more ACEs | N_obs_ (N_miss_) | 103 (0) | 82 (0) | 21 (0) |  |
| Yes | N (%) | 55 (53%) | 47 (57%) | 8 (38%) | 0.144 |
| ACE - physical abuse | N_obs_ (N_miss_) | 103 (0) | 82 (0) | 21 (0) |  |
| Yes | N (%) | 53 (51%) | 46 (56%) | 7 (33%) | 0.087 |
| ACE - sexual abuse | N_obs_ (N_miss_) | 103 (0) | 82 (0) | 21 (0) |  |
| Yes | N (%) | 8 ( 8%) | 7 ( 9%) | 1 ( 5%) | 1.000 |
| ACE - any abuse (physical or sexual) | N_obs_ (N_miss_) | 103 (0) | 82 (0) | 21 (0) |  |
| Yes | N (%) | 55 (53%) | 47 (57%) | 8 (38%) | 0.144 |
| Parental incarceration | N_obs_ (N_miss_) | 103 (0) | 82 (0) | 21 (0) |  |
| Yes | N (%) | 59 (57%) | 47 (57%) | 12 (57%) | 1.000 |
| *Physical punishment, partner violence or sexual assault |  |  |  |  |  |
| **Physical punishment or sexual assault |  |  |  |  |  |
|  | | | | | |

**Disability Outcome**

**Table S8: Conditions causing disability and differences by group**

| **Variable** | **Statistic** | **All (N = 103)** | **SHI (N = 82)** | **NoSHI (N = 21)** | **Fisher P** |
| --- | --- | --- | --- | --- | --- |
| Mental health complaints | N (%) | 73 (71%) | 59 (72%) | 14 (67%) | 0.788 |
| Anger | N (%) | 36 (35%) | 30 (37%) | 6 (29%) | 0.611 |
| Anxiety | N (%) | 28 (27%) | 23 (28%) | 5 (24%) | 0.789 |
| Depression | N (%) | 12 (12%) | 11 (13%) | 1 ( 5%) | 0.451 |
| Psychotic | N (%) | 5 ( 5%) | 5 ( 6%) | 0 ( 0%) | 0.580 |
|  | | | | | |

**Cognitive Function**

**Table S9: Cognitive function; z-scores are adjusted for age, years of education and delayed word memory score (*Lower values for overall cognitive impairment represent greater cognitive impairment)**

| **Variable** | **Statistic** | **All (N = 103)** | **SHI (N = 82)** | **NoSHI (N = 21)** | **P-value** |
| --- | --- | --- | --- | --- | --- |
| Cognitive function |  |  |  |  |  |
| Word memory immediate score | N_obs_ (N_miss_) | 103 (0) | 82 (0) | 21 (0) |  |
|  | Mean (SD) | 34.8 (3.8) | 34.6 (3.9) | 35.9 (3.4) |  |
|  | Range | (25.0, 40.0) | (25.0, 40.0) | (29.0, 40.0) | 0.177 |
| Word memory delayed score | N_obs_ (N_miss_) | 103 (0) | 82 (0) | 21 (0) |  |
|  | Mean (SD) | 36.1 (3.9) | 35.7 (4.1) | 37.6 (2.4) |  |
|  | Range | (23.0, 40.0) | (23.0, 40.0) | (33.0, 40.0) | 0.053 |
| Word memory consistency score | N_obs_ (N_miss_) | 103 (0) | 82 (0) | 21 (0) |  |
|  | Mean (SD) | 33.9 (4.4) | 33.6 (4.5) | 35.1 (3.6) |  |
|  | Range | (22.0, 40.0) | (22.0, 40.0) | (28.0, 40.0) | 0.167 |
| Symbol digit score | N_obs_ (N_miss_) | 103 (0) | 82 (0) | 21 (0) |  |
|  | Mean (SD) | 43.4 (10.2) | 42.7 (9.7) | 46.3 (11.6) |  |
|  | Range | (25.0, 76.0) | (25.0, 71.0) | (30.0, 76.0) | 0.141 |
| Symbol digit adjusted z-score | N_obs_ (N_miss_) | 102 (1) | 81 (1) | 21 (0) |  |
|  | Mean (SD) | 0.000 (1.000) | -0.067 (0.941) | 0.260 (1.190) |  |
|  | Range | (-2.380, 2.958) | (-2.380, 2.417) | (-1.858, 2.958) | 0.182 |
| List learning score | N_obs_ (N_miss_) | 103 (0) | 82 (0) | 21 (0) |  |
|  | Mean (SD) | 40.3 (9.6) | 39.9 (9.9) | 41.7 (8.3) |  |
|  | Range | (21.0, 63.0) | (21.0, 63.0) | (26.0, 52.0) | 0.440 |
| List learning adjusted z-score | N_obs_ (N_miss_) | 102 (1) | 81 (1) | 21 (0) |  |
|  | Mean (SD) | 0.000 (1.000) | -0.002 (1.040) | 0.009 (0.852) |  |
|  | Range | (-2.395, 2.340) | (-2.395, 2.340) | (-1.445, 1.262) | 0.963 |
| Trail Making Test part B score | N_obs_ (N_miss_) | 98 (5) | 79 (3) | 19 (2) |  |
|  | Mean (SD) | 97.3 (37.8) | 97.3 (37.5) | 97.1 (39.9) |  |
|  | Range | (43.0, 238.0) | (44.0, 238.0) | (43.0, 177.0) | 0.985 |
| Trail Making Test part B adjusted z-score | N_obs_ (N_miss_) | 97 (6) | 78 (4) | 19 (2) |  |
|  | Mean (SD) | 0.000 (1.000) | -0.021 (0.989) | 0.087 (1.068) |  |
|  | Range | (-2.006, 3.053) | (-2.006, 3.053) | (-1.495, 1.963) | 0.676 |
| Verbal fluency animals score | N_obs_ (N_miss_) | 102 (1) | 81 (1) | 21 (0) |  |
|  | Mean (SD) | 19.6 (5.2) | 20.1 (5.1) | 17.7 (5.1) |  |
|  | Range | (9.0, 36.0) | (9.0, 36.0) | (9.0, 32.0) | 0.059 |
| Verbal fluency animals adjusted z-score | N_obs_ (N_miss_) | 101 (2) | 80 (2) | 21 (0) |  |
|  | Mean (SD) | 0.000 (1.000) | 0.116 (0.980) | -0.440 (0.973) |  |
|  | Range | (-2.191, 3.066) | (-2.191, 3.066) | (-1.792, 2.294) | 0.023 |
| Verbal fluency letters score | N_obs_ (N_miss_) | 101 (2) | 80 (2) | 21 (0) |  |
|  | Mean (SD) | 27.6 (10.4) | 27.5 (10.2) | 27.8 (11.2) |  |
|  | Range | (9.0, 58.0) | (9.0, 58.0) | (10.0, 51.0) | 0.922 |
| Verbal fluency letters adjusted z-score | N_obs_ (N_miss_) | 101 (2) | 80 (2) | 21 (0) |  |
|  | Mean (SD) | 0.000 (1.000) | 0.014 (0.985) | -0.052 (1.080) |  |
|  | Range | (-1.812, 2.707) | (-1.812, 2.707) | (-1.627, 2.237) | 0.789 |
| Overall cognitive impairment (adjusted z-score)* | N_obs_ (N_miss_) | 102 (1) | 81 (1) | 21 (0) |  |
|  | Mean (SD) | 0.000 (1.000) | 0.023 (0.982) | -0.090 (1.087) |  |
|  | Range | (-2.445, 2.274) | (-2.445, 2.274) | (-1.901, 1.993) | 0.645 |
|  | | | | | |

**Table S10: Executive function on the DEX Questionnaire**

| **Variable** | **Statistic** | **All (N = 103)** | **HI (N = 82)** | **NoSHI (N = 21)** | **P-Value** |
| --- | --- | --- | --- | --- | --- |
| Self-completed | N_obs_ (N_miss_) | 102 (1) | 81 (1) | 21 (0) |  |
|  | Mean (SD) | 35 (16) | 37 (15) | 26 (14) |  |
|  | Range | (2, 71) | (7, 71) | (2, 52) | 0.006 |
| Independently-completed | N_obs_ (N_miss_) | 82 (21) | 64 (18) | 18 (3) |  |
|  | Mean (SD) | 25 (17) | 26 (17) | 22 (15) |  |
|  | Range | (0, 69) | (0, 69) | (2, 57) | 0.356 |

**Comparison of Cognitive Test Scores with Test Norms**

Published norms for the general population, stratified where available for age, education and gender, were used to create z-scores for each individual and from these mean deviation from the norms are presented in table A7.

**Table S11: Comparison between cognitive test scores and published test norms**

| **Test** | **Stratification of**  **Test Norm** | **Test Norm (Mean; SD)** | **Juveniles in prison (N=102); Mean**  **Z-score difference** | **P value** |
| --- | --- | --- | --- | --- |
| **Symbol Digit Modalities Test^1^** | Age 15-19;  education, gender | 52.8; 12.1 | -0.78 | 0.22 |
| **Auditory Verbal Learning Test^2^** | Age 18-30 | 54.8; 7.7 | -1.88 | 0.06 |
| **Trail Making Test B^3^** | Age 18-24 | 49.0; 12.7 | 3.79 | <0.001 |
| **Verbal Fluency (letters)^4^** | Education; gender | 36.9; 9.8 | -0.95 | 0.34 |

1. Kiely KM, Butterworth P, Watson N et al (2014). The Symbol Digit Modalities Test: Normative Data from a Large Nationally Representative Sample of Australians. *Archives of Clinical Neuropsychology*, 29; 767–775
2. Coughlan AK & Hollows SE. The Adult Memory and Information Processing Battery Test Manual. Psychology Department, University of Leeds, Leeds, UK 1985.
3. Tombaugh T. Trail Making Test A and B: Normative data stratified by age and education. Archives of *Clin Neuropsychol* 2004: 19:203-214.
4. Ruff R, Light R, Parker S et al. Benton Controlled Oral Word Association Test: reliability and updated norms. *Archiv Clin Neuropsychol* 1996: 11(4): 329-338.
