# Supplementary material for: Associations between significant head injury in male juveniles in prison in Scotland UK and cognitive function, disability and crime: A cross sectional study
Source: PLoS One. 2023 Jul 12;18(7):e0287312. doi: 10.1371/journal.pone.0287312 (PMC10337871; doi:10.1371/journal.pone.0287312)
Supplement: S3 File — (DOCX) [file pone.0287312.s003.docx]

**S3 Supplementary Figures**

**Disability**

**Figure S1: current risk factors for HI-attributed disability. Odds ratios and 95% confidence intervals for SHI vs No-SHI groups**

**
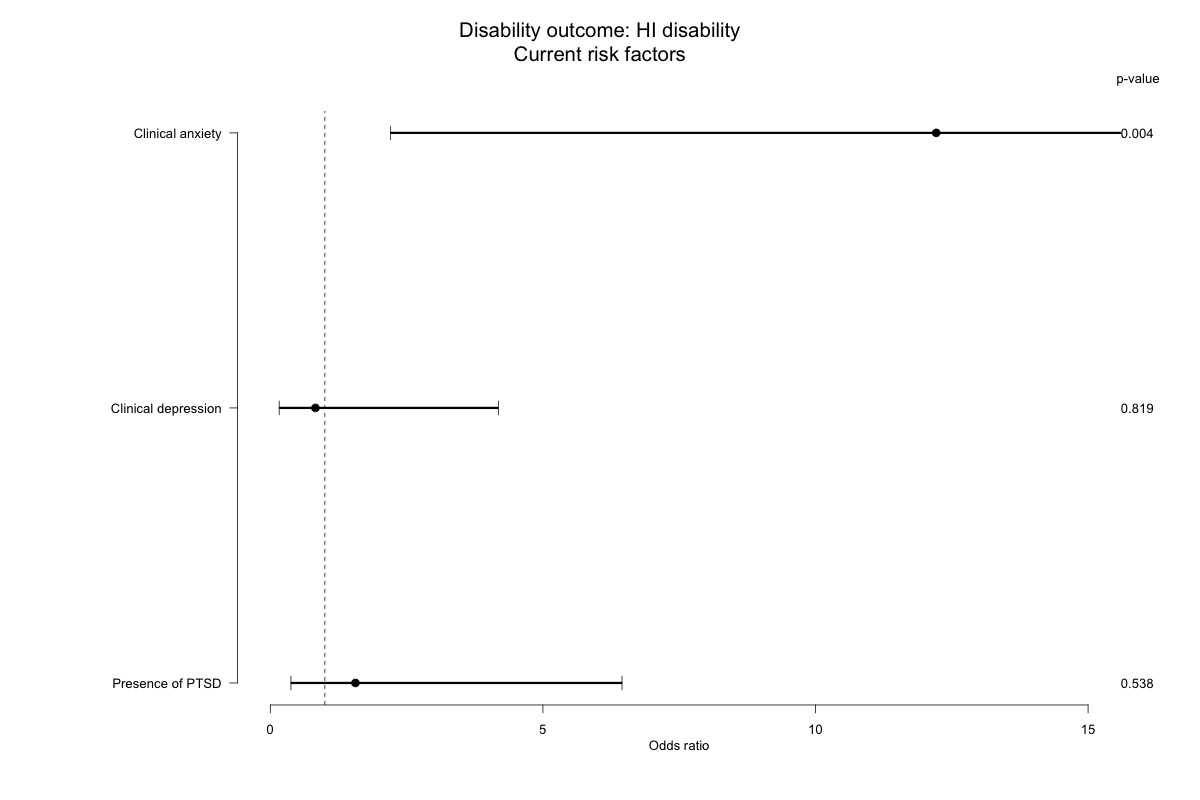
**

**Figure S2: Historical risk factors for HI-attributed disability for SHI vs No-SHI groups Odds ratios and 95% confidence intervals**

**
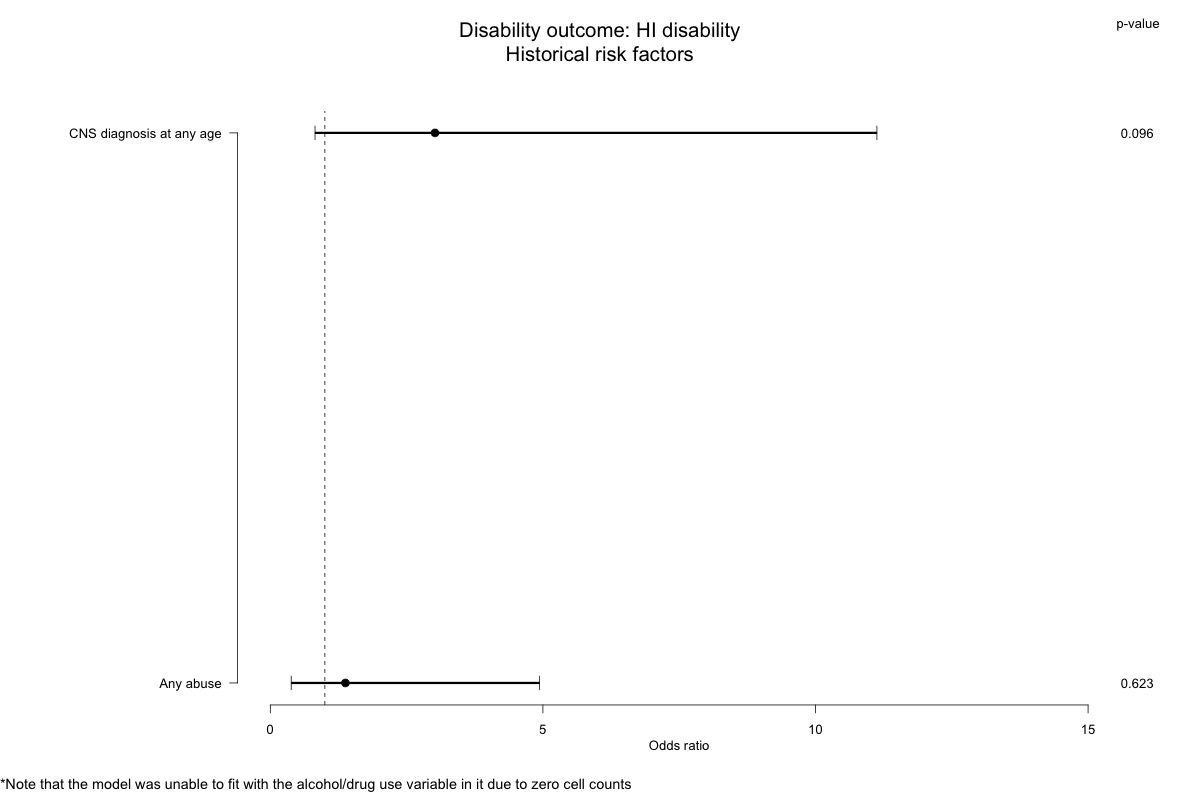
**

**Figure S3: Current risk factors for disability of any cause for SHI vs No-SHI groups Odds ratios and 95% confidence intervals unadjusted (left) and adjusted (right)**

**
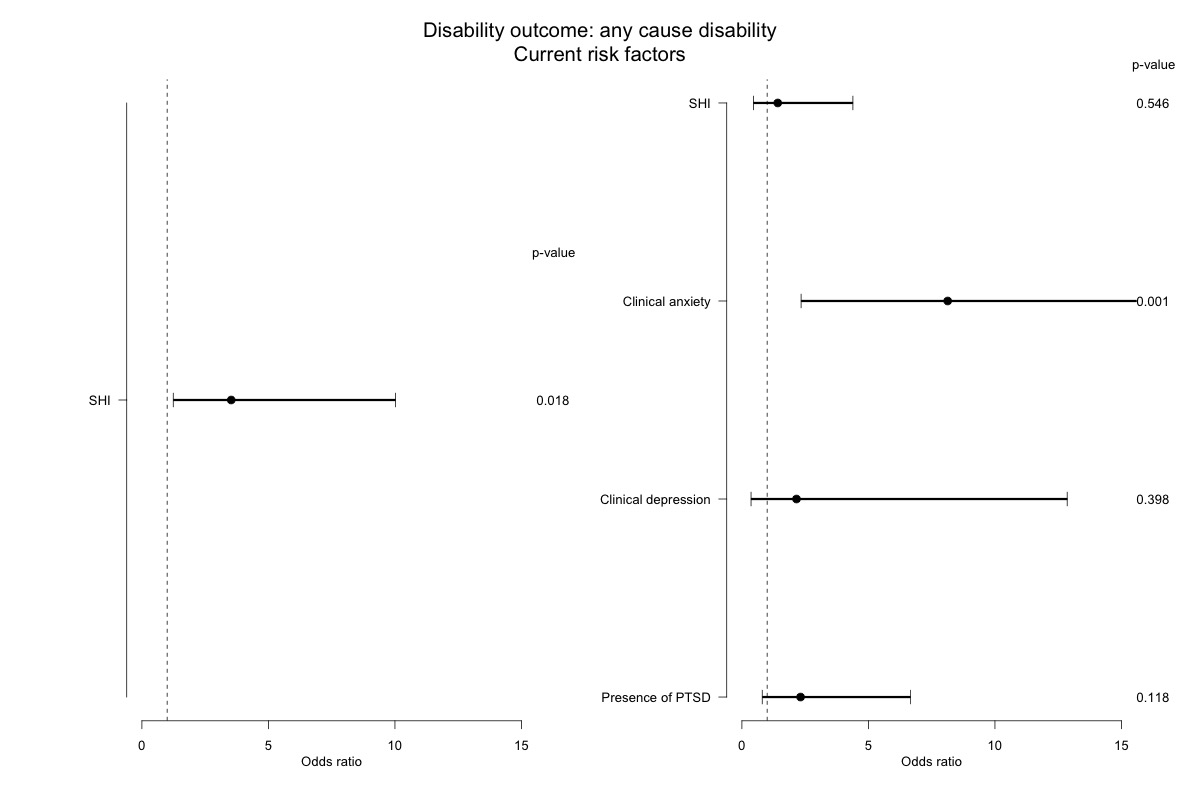
**

**Figure S4a: Historical risk factors for disability of any cause for SHI vs No-SHI groups, including clinically problematic substance use. Odds ratios and 95% confidence intervals unadjusted (left) and adjusted (right)**

**
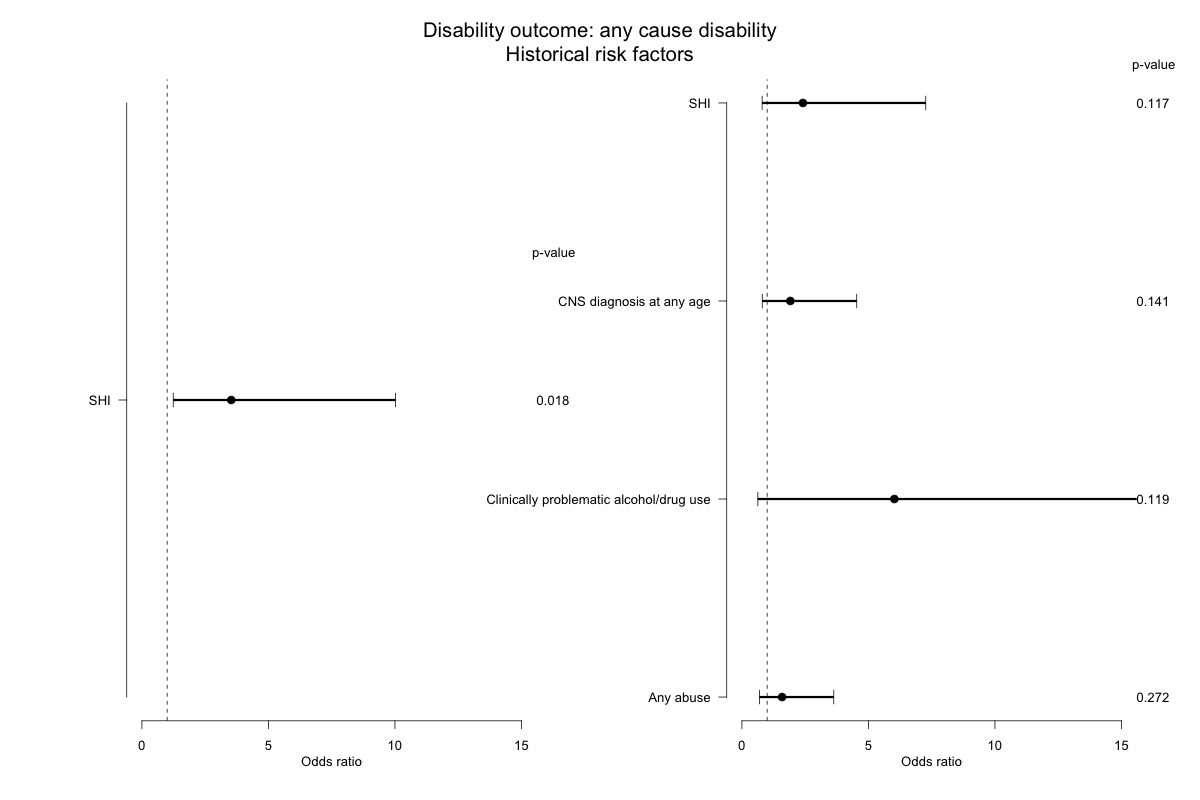
**

**Figure S4b: Historical risk factors for disability of any cause for SHI vs No-SHI groups, including self-report of problematic substance use. Odds ratios and 95% confidence intervals unadjusted (left) and adjusted (right)including self-report of problematic substance use.**

**
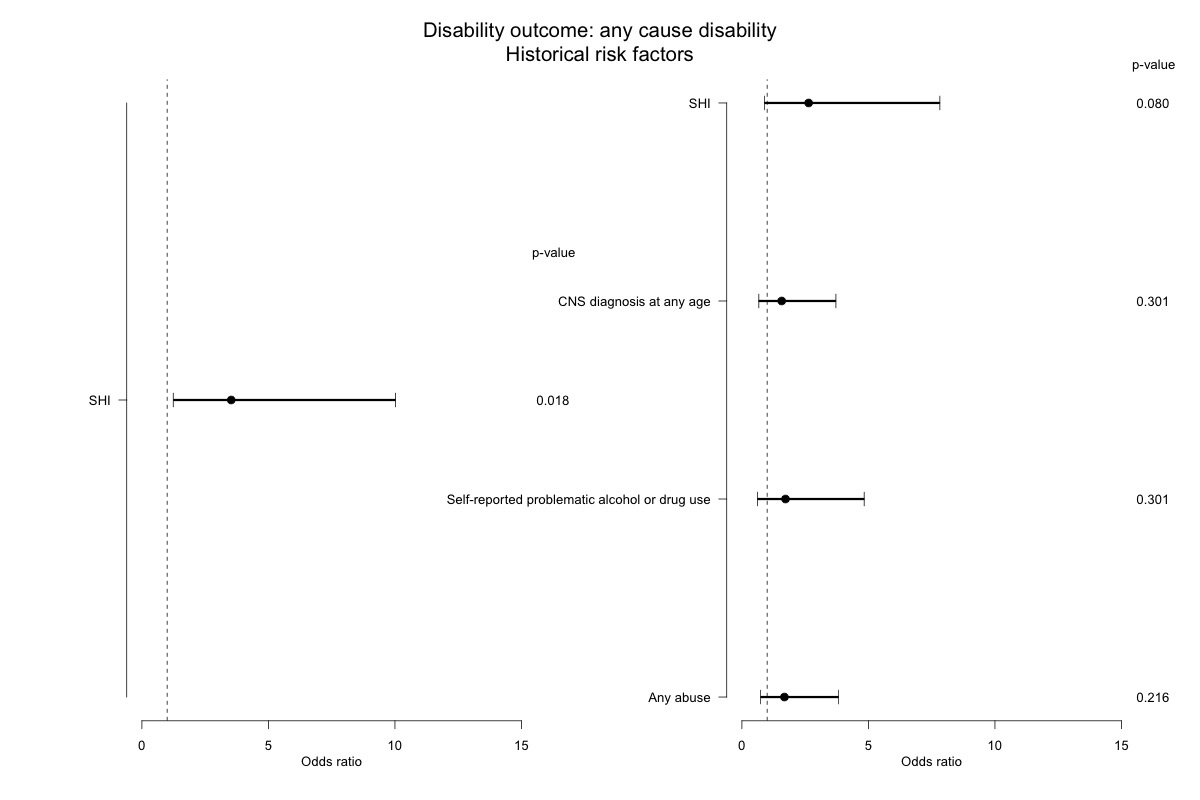
**

**Cognitive Function**

**Figure S5: Individual cognitive tests by group. Box and whisker plots for raw scores (upper) and z-scores (lower)**

**
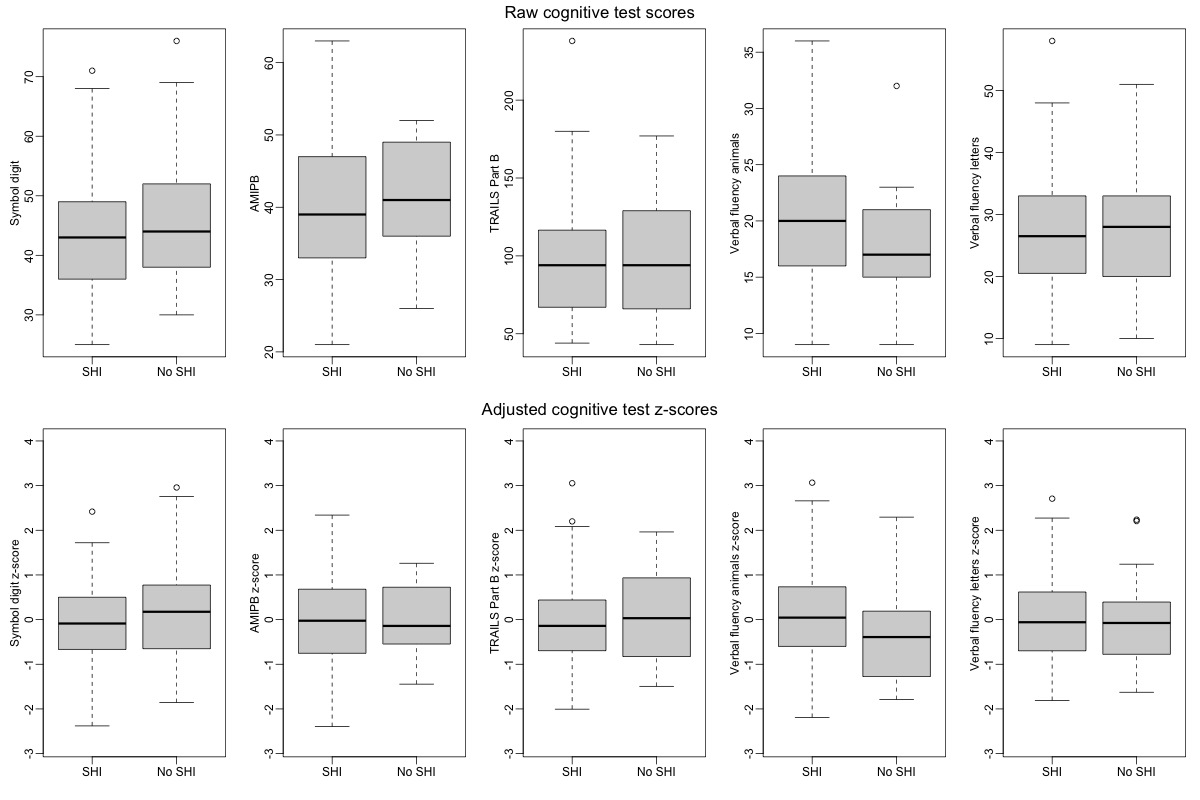
**

**Figure S6: Box and whisker plots for overall cognitive z-scores by group**

**
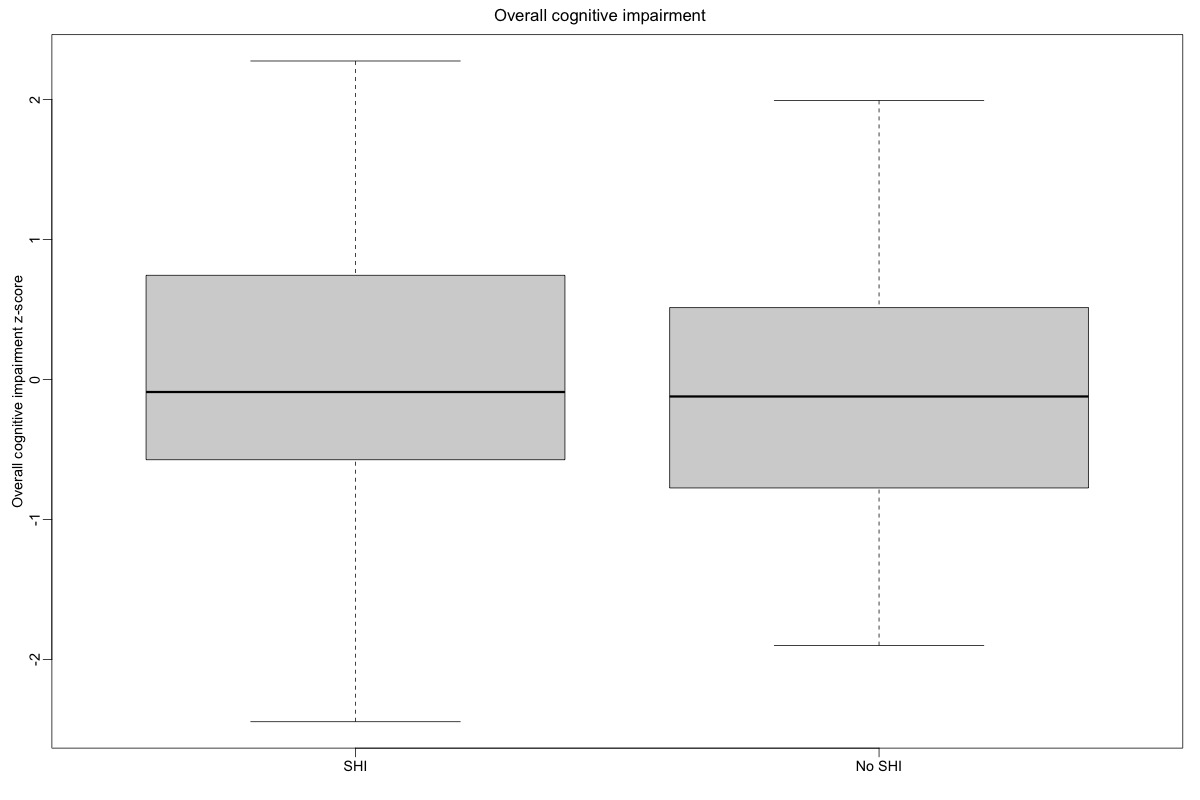
**

Current clinical anxiety was associated with a marginal decrease in cognitive function score (mean difference -0.54, 95% CI -1.03,-0.06). Current PTSD was marginally associated with a small increase in cognitive function score (mean difference 0.48, 95% CI 0.03-0.92). However, univariable analysis of each of these variables indicated no evidence of association (anxiety mean difference -0.27, 95% CI -0.68,0.16; PTSD 0.34, 95% CI -0.07,0.76) suggesting these may not be robust effects. See figure S7.

**Figure S7: Current risk factors for poorer cognitive function in SHI and No-SHI groups. Estimated mean difference between groups with 95% confidence intervals, unadjusted (left) and adjusted (right); (lower values = poorer test performance)**

**
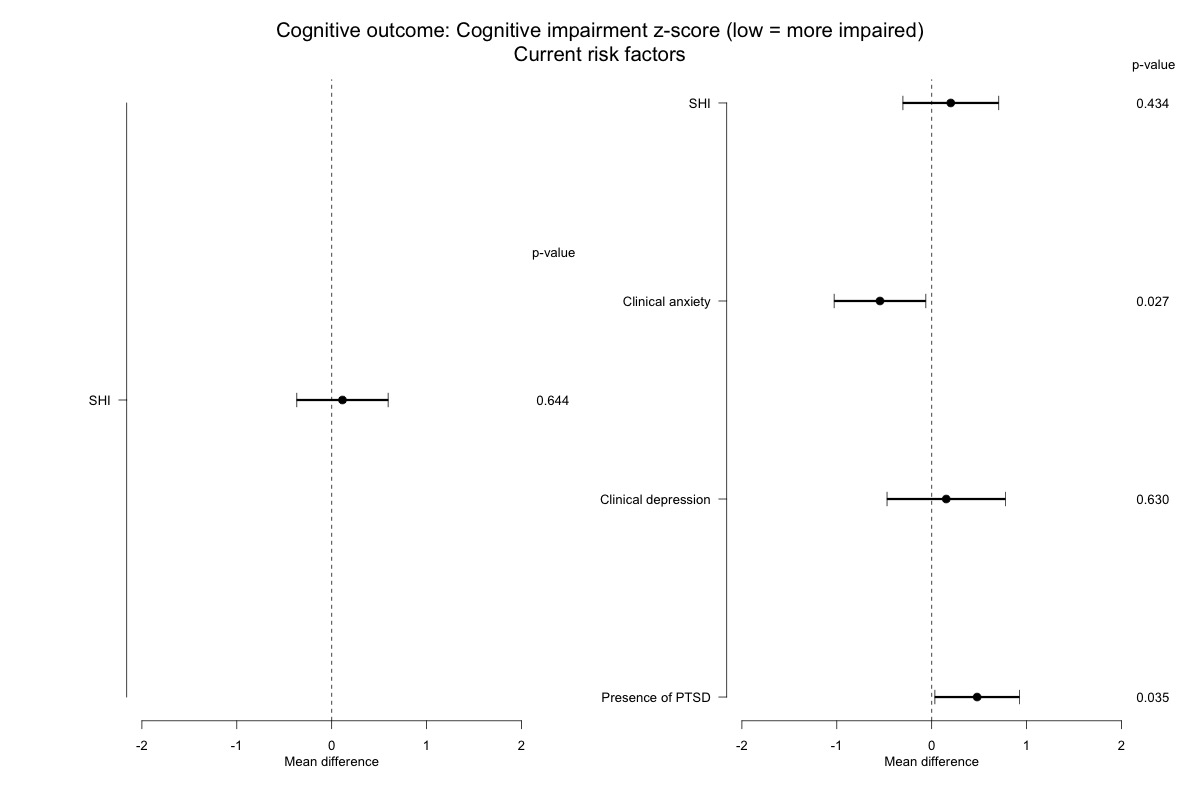
**

**Figure S8a: Historical risk factors for poorer cognitive function in SHI and No-SHI groups, including clinically problematic substance use. Estimated mean difference between groups with 95% confidence intervals, unadjusted (left) and adjusted (right); (lower values = poorer test performance)**

**
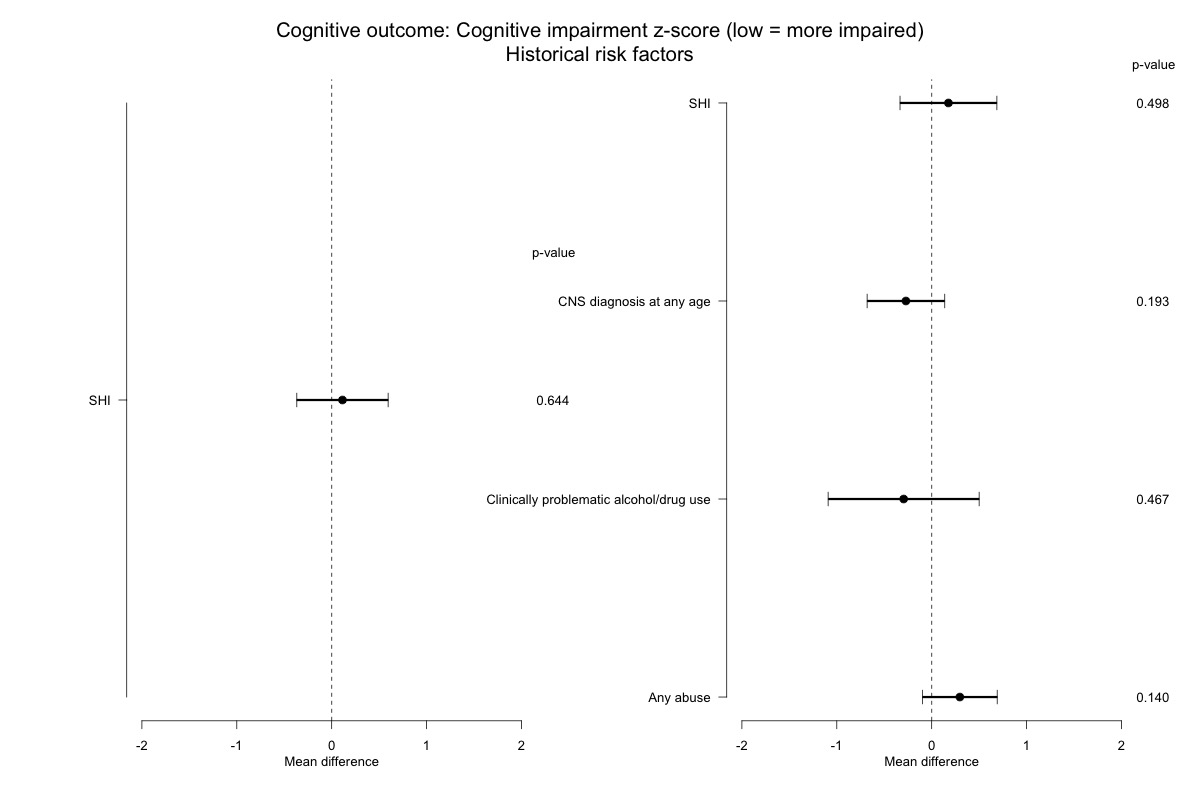
**

**Figure S8b: Historical risk factors for poorer cognitive function in SHI and No-SHI groups, including self-reported problematic substance use. Estimated mean difference between groups with 95% confidence intervals, unadjusted (left) and adjusted (right); (lower values = poorer test performance)**

**
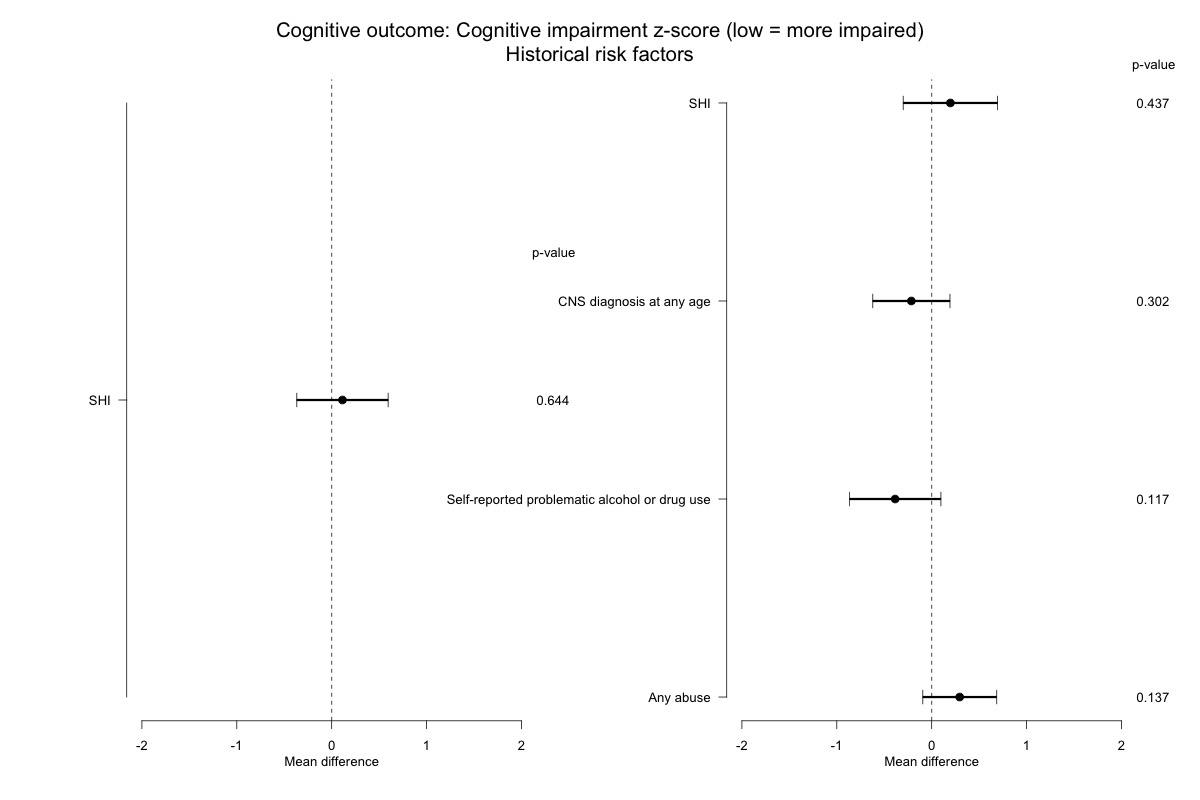
**

**Offending**

The total number of ACEs was marginally associated with more convictions in univariable analysis (For each increase of 1 ACE, Rate Ratio increased number of convictions by 1.1; 95% CI 1.0-1.3). Adjusting for SHI made no difference to this relationship. There was no statistically significant association between number of ACEs and violent offences (OR=1.13; 95% CI 0.91-1.39).

**Figure S9: Current risk factors for number of convictions. Rate ratios and 95% confidence intervals for SHI vs No-SHI groups unadjusted (left) and adjusted (right)**

**
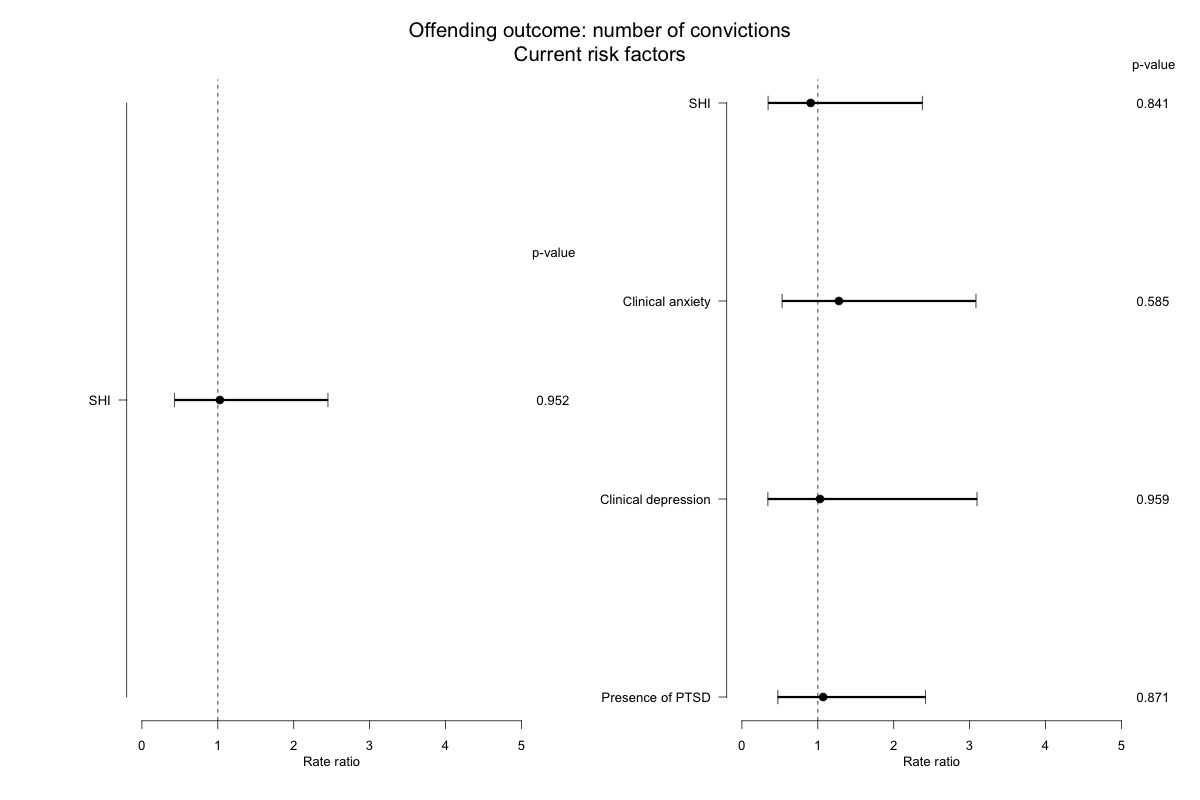
**

**Figure S10a: Historical risk factors for number of convictions including clinically problematic substance abuse. Rate ratios and 95% confidence intervals for SHI vs No-SHI groups unadjusted (left) and adjusted (right)**

**
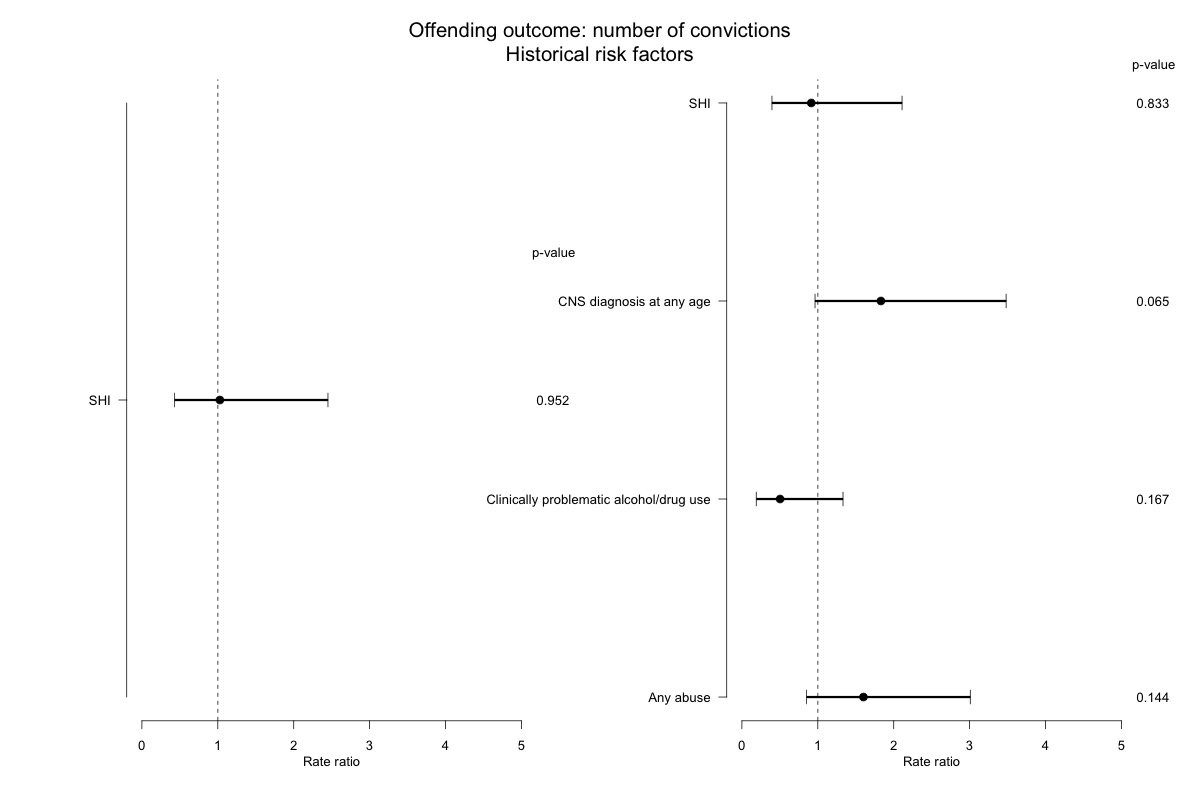
**

**Figure S10b: Historical risk factors for number of convictions including self-reported problematic substance abuse. Rate ratios and 95% confidence intervals for SHI vs No-SHI groups unadjusted (left) and adjusted (right)**

**
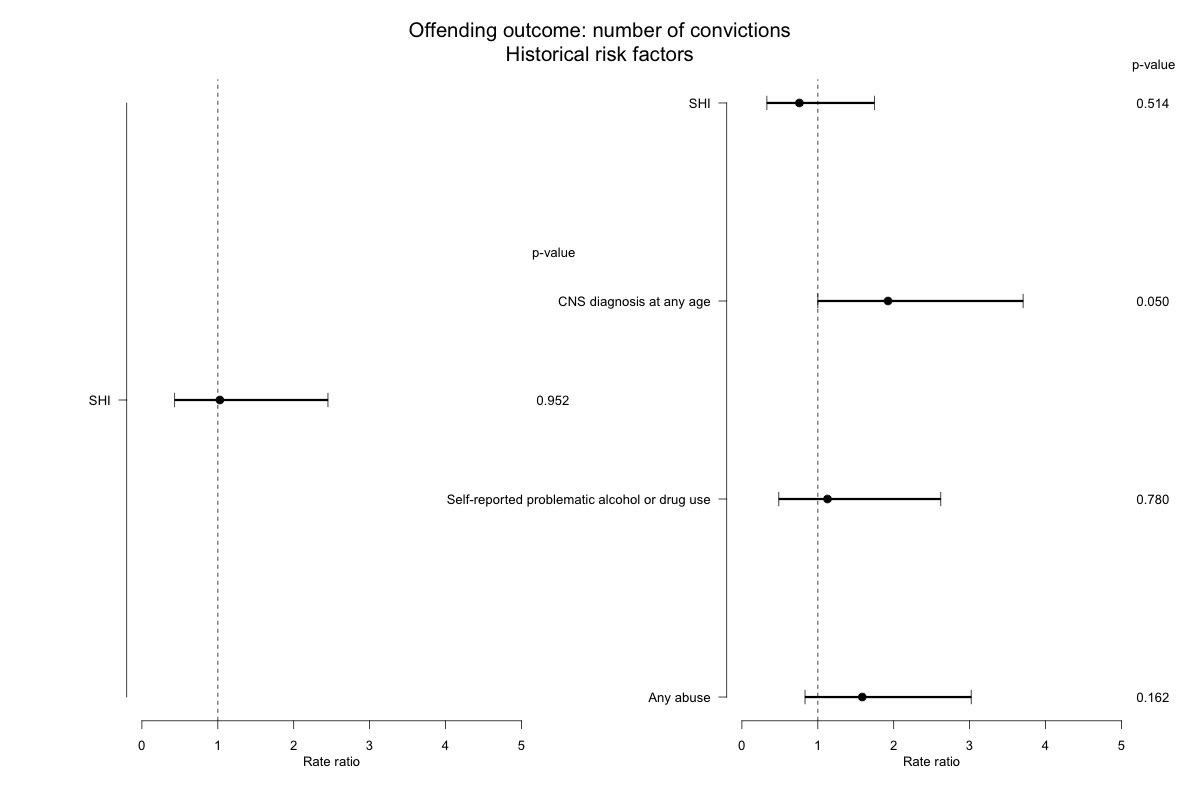
**

**Figure S11: Current risk factors for longest length of sentence. Rate ratios and 95% confidence intervals for SHI vs No-SHI groups unadjusted (left) and adjusted (right)**

**
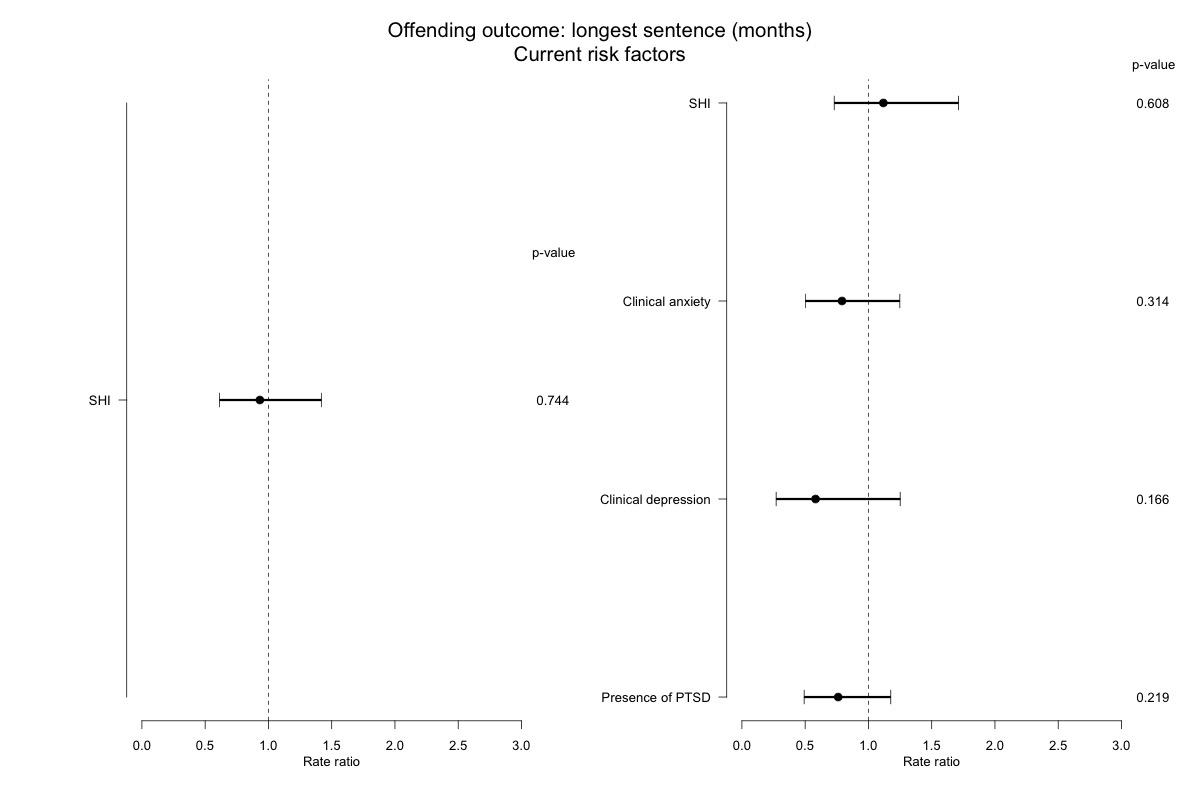
**

**Figure S12a: Historical risk factors for longest length of sentence including clinically problematic substance abuse. Rate ratios and 95% confidence intervals for SHI vs No-SHI groups unadjusted (left) and adjusted (right)**

**
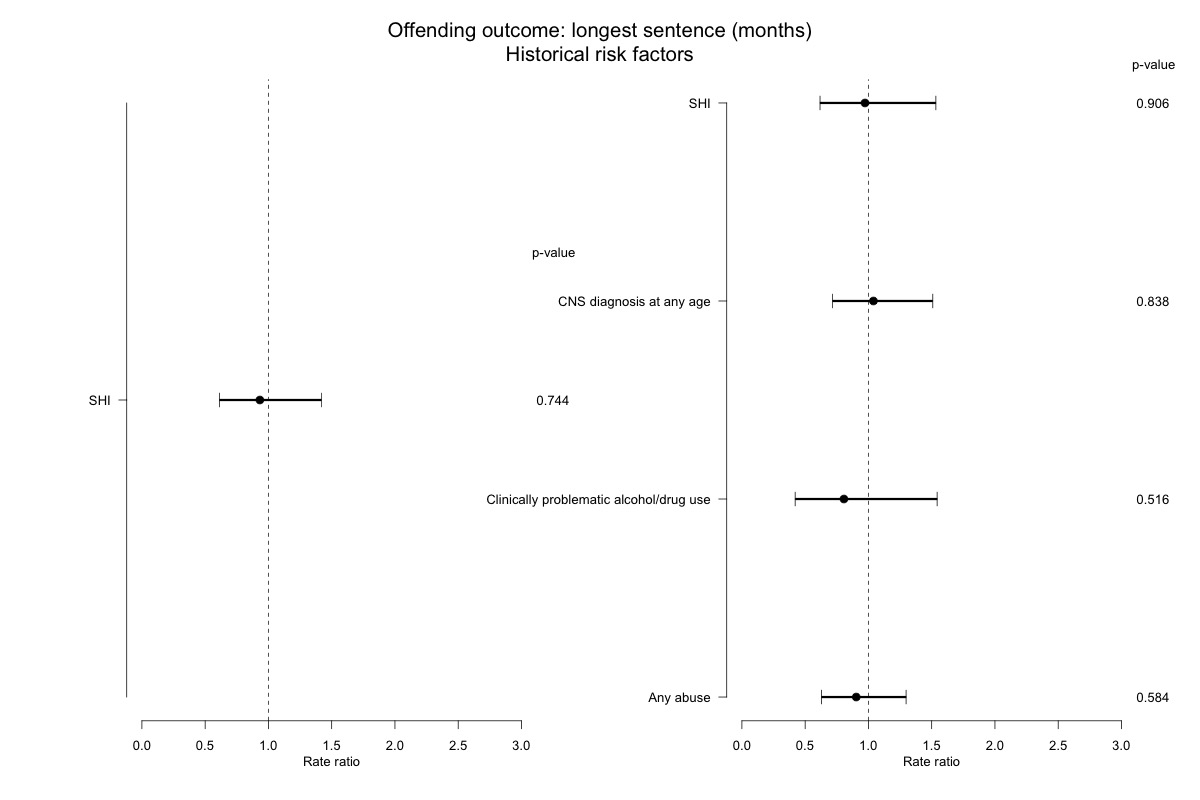
**

**Figure S12b: Historical risk factors for longest length of sentence including self-reported problematic substance abuse. Rate ratios and 95% confidence intervals for SHI vs No-SHI groups unadjusted (left) and adjusted (right)**

**
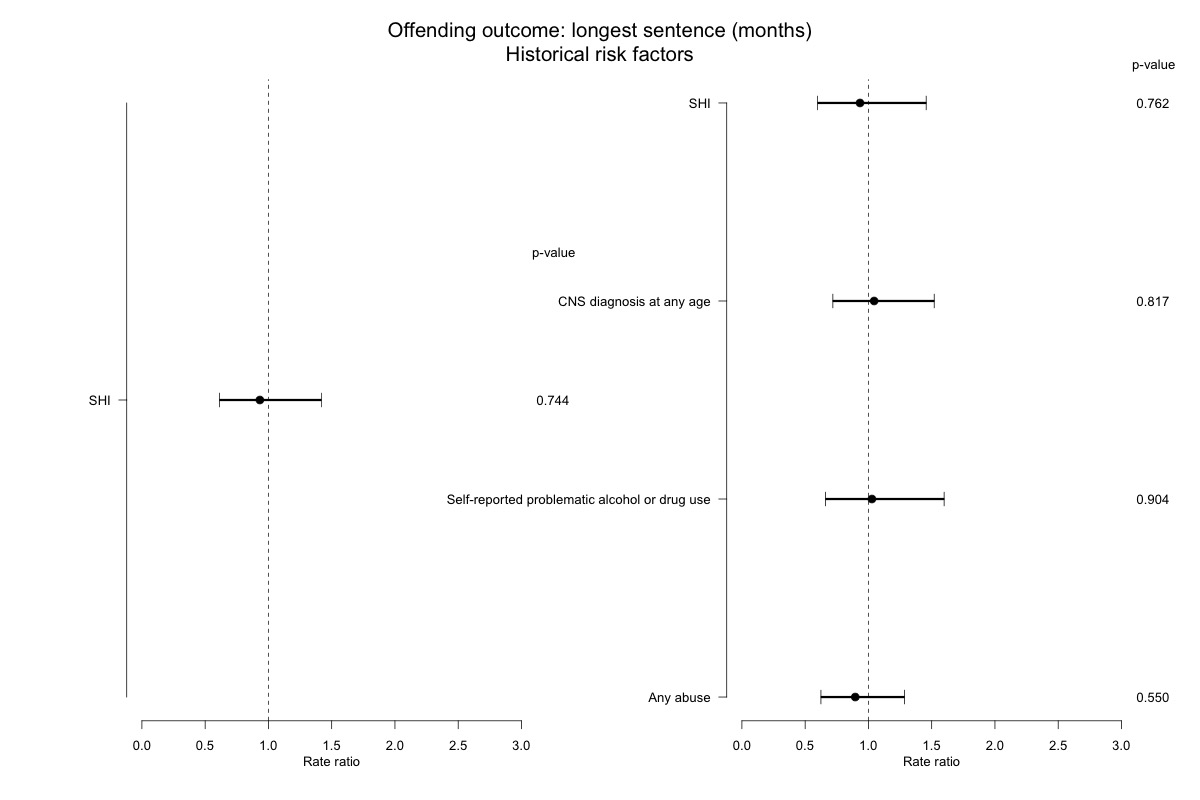
**

**Figure S13: Current risk factors for violent/non-violent offending. Odds ratios and 95% confidence intervals for SHI vs No-SHI groups unadjusted (left) and adjusted (right)**

**
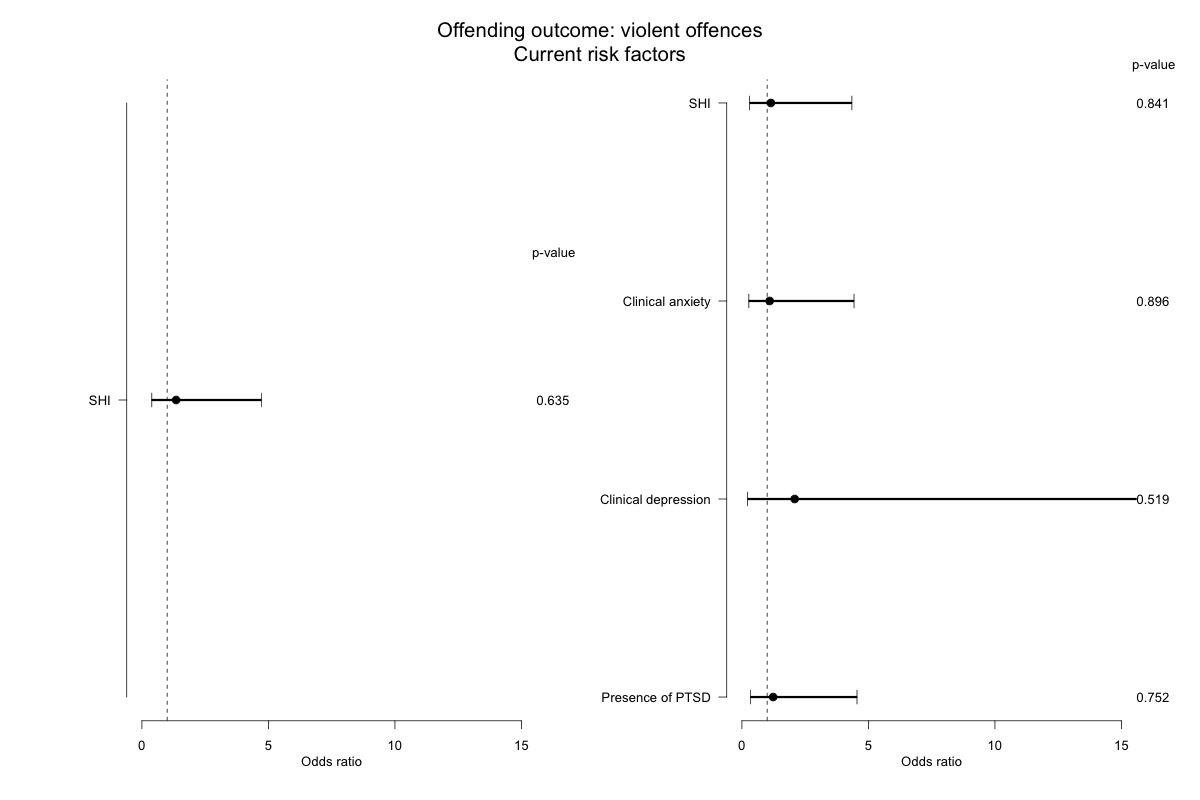
**

**Figure S14a: Historical risk factors for violent/non-violent offending including clinically problematic substance abuse. Odds ratios and 95% confidence intervals for SHI vs No-SHI groups unadjusted (left) and adjusted (right)**

**
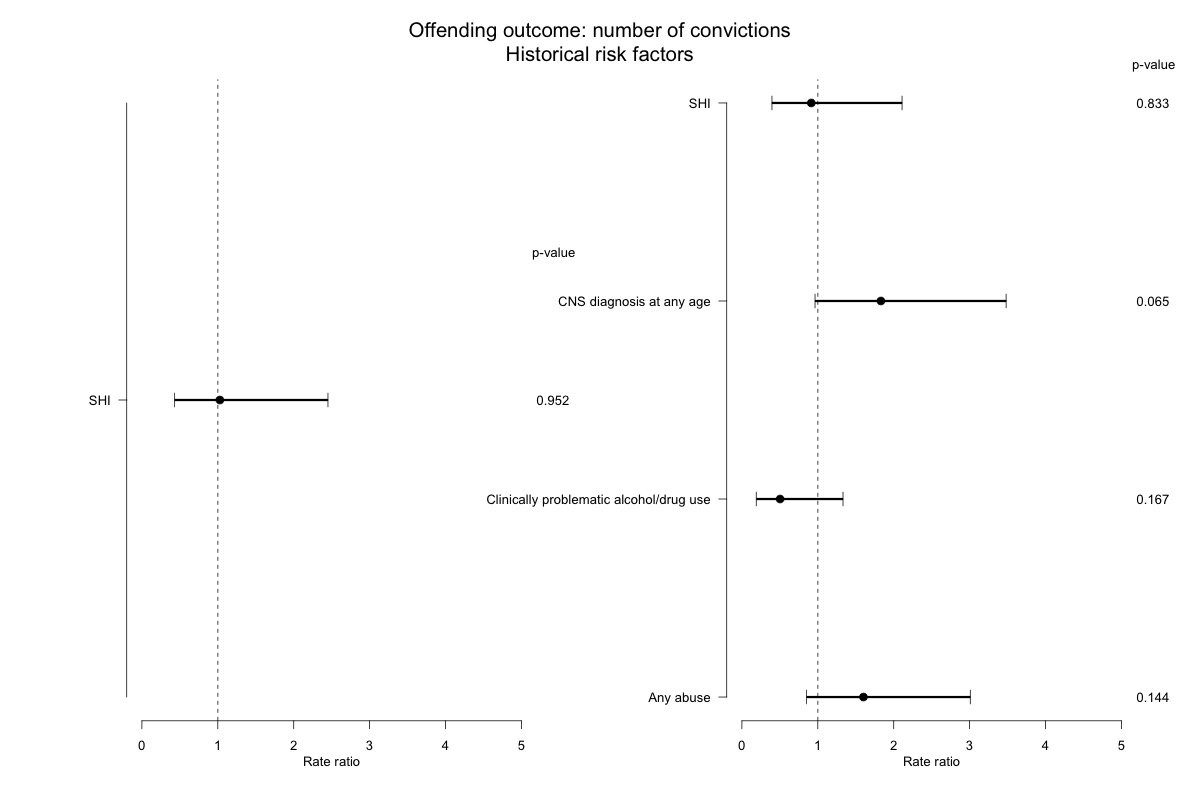
**

**Figure S14b: Historical risk factors for violent/non-violent offending including self-reported problematic substance abuse. Odds ratios and 95% confidence intervals for SHI vs No-SHI groups unadjusted (left) and adjusted (right)**

**
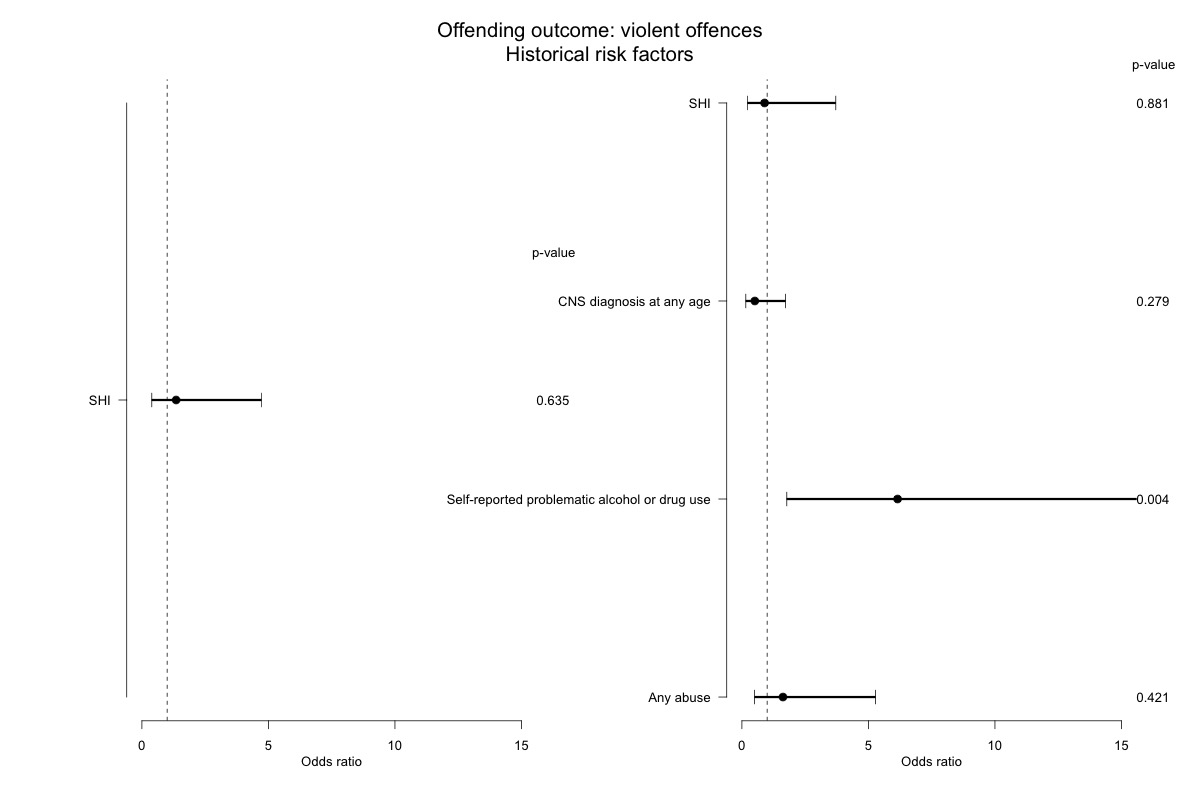
**
