# Supplementary material for: Associations between significant head injury in male juveniles in prison in Scotland UK and cognitive function, disability and crime: A cross sectional study
Source: PLoS One. 2023 Jul 12;18(7):e0287312. doi: 10.1371/journal.pone.0287312 (PMC10337871; doi:10.1371/journal.pone.0287312)
Supplement: Attachment — Submitted filename: MSReview-PONE-D-22-23246.docx [file pone.0287312.s004.docx]

MS Review for *PLOS ONE*

MS ID: PONE-D-22-23246

TITLE: "Associations between Significant Head Injury in Male Juveniles in Prison in Scotland UK and Cognitive Function, Disability and Crime: A Cross sectional study"

AUTHORS: T M McMillan, J McVean, H Aslam, S J E Barry

**Comments to the authors**

This is a well written study presenting the results of original research that has not, to my best knowledge, been published elsewhere. All other five criteria for publication on PLOS ONE are met, although I would suggest a few minor revisions to help improve reproducibility and clarity.

It would be useful to describe the procedure in a little more detail. In particular, on page six, it is mentioned that the assessments “were carried out by a final year doctorate in clinical psychology trainee and an experienced researcher. Does this mean that both assessors were present for each individual assessment, or each assessor worked with different participants. If the latter, could the authors describe what measures were taken to ensure reliability (e. g. training, inter-rater reliability procedures and estimates)?

For interest, what proportion of participants required an assessment over two sessions? This aspect can have practical implications when carrying out studies like these in “live” environments, and having an understanding of how frequent, or not, more resources are required may help other researchers better plan future studies.

Is the median number of head injuries of five reported on page 11 for the whole SHI group, or only for the 23 who had more than two injuries? If the latter, could the authors also report the median number of injuries within the SHI group overall?

Again, for interest, what was the question used to obtain information about experiences of anoxia due to strangulation?

On page 14, the authors report on the history of physical and sexual abuse, including “adult sexual abuse”. Although the interpretation would not necessarily change, should the denominator for those percentages not be calculated out of the total number of adults in the sample (i. e. only those aged ≥ 18), rather than the full sample?

Please provide more detail about how the attribution of disability on the GODS was achieved. Given the characteristics of the injuries in the SHI group, and more generally in this population (i. e. multiple, mild, and often acquired over an extended period of time), could the authors comment on the possible limitation in terms of reliability and validity of these attributions of disability specifically to head injuries?

Please revise table 4, page 17. The percentages shown on column three for the “Disability from SHI” data do not match with the total *N* shown on the top of that column (i. e. 70/103 is 68%, not 85%, etc.), and the same results are reported twice (on column three and column four). Should the percentages for this sub-sample not be calculated out of the total of people with head injuries (*N* = 82) and then out of the total of those who specifically attributed disabilities to head injury (i. e. *N* = 11), rather than the overall sample?

Could the authors calculate what the scores on the DEX for the 18 participants who were not rated by prison officers were, and explore whether these are comparable to the rest of the sample (in other words, is there a chance that prison officers happened not to provide ratings for those scoring themselves higher on this measure)?

It may be useful to add one or two citations about the limitations of using structured assessments and of assessing people with brain injuries and dysexecutive difficulties in structured environments (p. 23, second paragraph), as this would help readers less familiarised with the literature in this field to get more acquainted with the evidence-base for this challenge.

**Minor edits**

p. 7 – Expand the GODS acronym at first use.

p. 8 – Consider replacing “on more than two occasions” with “on three or more occasions” for clarity and consistency.

p. 10 and throughout – sometimes proportions are reported percentage first, then number ratios, other times the other way around (e. g. p. 10). Please use the same order throughout for simplicity.

pp. 11-12 – Consider referring to “head injuries” (rather than “head injury”) when describing multiple or repeated occurrences of injury.

p. 18 – Please ensure that all p-values are reported consistently. For example, the p-value for the difference between prison officers’ reports on the DEX by group is missing.
