# Supplementary material for: Associations between significant head injury in male juveniles in prison in Scotland UK and cognitive function, disability and crime: A cross sectional study
Source: PLoS One. 2023 Jul 12;18(7):e0287312. doi: 10.1371/journal.pone.0287312 (PMC10337871; doi:10.1371/journal.pone.0287312)
Supplement: Attachment — Submitted filename: Response to Reviewers_Dec22docx.docx [file pone.0287312.s005.docx]

**Response to Reviewers**

**PONE-D-22-23246**
Associations between Significant Head Injury  in Male Juveniles in Prison in Scotland UK and Cognitive Function, Disability and Crime: A Cross sectional study

**Editors Comments**
In addition to the reviewer requests, please also ensure that you include the following information on resubmission:

a) A complete ethics statement in the Methods section, including the names of both approving bodies and any approval numbers

We have added to the approvals section on page 10.

b) Information on data access restrictions, such as where and how other interested researchers are able to apply to the ethics committee or data access committee. Please include this information in the Data availability statement in the online submission form

We have added to the online data availability statement

**Reviewer #1**: This is a well written study presenting the results of original research that has not, to my best knowledge, been published elsewhere. All other five criteria for publication on PLOS ONE are met, although I would suggest a few minor revisions to help improve reproducibility and clarity.

It would be useful to describe the procedure in a little more detail. In particular, on page six, it is mentioned that the assessments “were carried out by a final year doctorate in clinical psychology trainee and an experienced researcher. Does this mean that both assessors were present for each individual assessment, or each assessor worked with different participants. If the latter, could the authors describe what measures were taken to ensure reliability (e. g. training, inter-rater reliability procedures and estimates)?

Changes have been made on page 6 and information on assessor training in the Supplement on page 26.

For interest, what proportion of participants required an assessment over two sessions? This aspect can have practical implications when carrying out studies like these in “live” environments, and having an understanding of how frequent, or not, more resources are required may help other researchers better plan future studies.

We do not have an exact figure. It was around 10%. We have added two sentences in the Discussion p 23/24.

Is the median number of head injuries of five reported on page 11 for the whole SHI group, or only for the 23 who had more than two injuries? If the latter, could the authors also report the median number of injuries within the SHI group overall?

It is for the 23; we think this is clearly indicated in the text already. We have added information on the median number in the SHI group overall.

Again, for interest, what was the question used to obtain information about experiences of anoxia due to strangulation?

This was derived in two ways. From responses on the OSU-TBI regarding loss of consciousness -when a participant indicated that this had occurred they were asked for information about cause. Second the “Other CNS System Compromise questions given as part of the OSU-TBI assessment asks about history of ‘oxygen deprivation (anoxia)’. This has been added to procedure on page 7.

On page 14, the authors report on the history of physical and sexual abuse, including “adult sexual abuse”. Although the interpretation would not necessarily change, should the denominator for those percentages not be calculated out of the total number of adults in the sample (i. e. only those aged ≥ 18), rather than the full sample?

We have altered the text to read ‘occurring when aged 16 or over’ rather than adult on pages 14 and 16 and in the table in the supplement on page 6. We have also added further information on the age of participants on page 10. This reflects minimum age of admission to prison as a juvenile and is more easily justified given differences/complexities in definition if ‘adulthood’ internationally.

Please provide more detail about how the attribution of disability on the GODS was achieved. Given the characteristics of the injuries in the SHI group, and more generally in this population (i. e. multiple, mild, and often acquired over an extended period of time), could the authors comment on the possible limitation in terms of reliability and validity of these attributions of disability specifically to head injuries?

The assessment of whether disability was present, how severe it might be and whether attributable to head injury followed the administration process of the Glasgow Outcome Scales which have been extensively validated. The structured interview investigates whether there is disability and if so whether this is attributable to head injury by clarifying timelines in relation to the head injury, reported sequelae, and participant attribution of symptoms to HI (if offered). There is also cross-referencing with other information, such as that obtained from the OSU-TBI to prompt during the interview and help to assist in judgements. We do not feel it appropriate to detail this in the paper as it is a general principle underpinning outcome assessments using the Glasgow Scales and follows their guidelines. We have though added to the Methods on page 8 and Discussion with regard to multiple mild injuries on pages 24/25.

Please revise table 4, page 17. The percentages shown on column three for the “Disability from SHI” data do not match with the total N shown on the top of that column (i. e. 70/103 is 68%, not 85%, etc.), and the same results are reported twice (on column three and column four). Should the percentages for this sub-sample not be calculated out of the total of people with head injuries (N = 82) and then out of the total of those who specifically attributed disabilities to head injury (i. e. N = 11), rather than the overall sample?

We see this could be misleading and have deleted the ‘total’ column from table 4. The figures in the table were in fact correct.

Could the authors calculate what the scores on the DEX for the 18 participants who were not rated by prison officers were, and explore whether these are comparable to the rest of the sample (in other words, is there a chance that prison officers happened not to provide ratings for those scoring themselves higher on this measure)?

A sentence has been added at the end of the cognitive function section on page 18.

It may be useful to add one or two citations about the limitations of using structured assessments and of assessing people with brain injuries and dysexecutive difficulties in structured environments (p. 23, second paragraph), as this would help readers less familiarised with the literature in this field to get more acquainted with the evidence-base for this challenge.

A citation to a review has been added on page 23.

**Minor edits**
p. 7 – Expand the GODS acronym at first use. Done (p7 last para)

p. 8 – Consider replacing “on more than two occasions” with “on three or more occasions” for clarity and consistency. No change made -this is the description we have used in previous studies.

p. 10 and throughout – sometimes proportions are reported percentage first, then number ratios, other times the other way around (e. g. p. 10). Please use the same order throughout for simplicity. Done

pp. 11-12 – Consider referring to “head injuries” (rather than “head injury”) when describing multiple or repeated occurrences of injury. I have left this as is– not to assume that during an event there were several head injuries.

p. 18 – Please ensure that all p-values are reported consistently. For example, the p-value for the difference between prison officers’ reports on the DEX by group is missing.

We have added this p-value and a small number of others that were missing.

**Reviewer #2:** this is a welcome and helpful addition to the literature. a strong study, with good measures, of brain trauma in young offenders. the findings were consistent and enhance previous work. The study adds that brain injury is linked to greater issues in anxiety and other issues (eg abuse/neglect) and ongoing issues in self-regulation. these are important findings for better services around such young people. there is a very good discussions, which is timely, on such young people being at a crossroads, before greater violence becomes an issue. the study is well conceived, well run, and has important findings.

In future it would be good to try and look more at the gradings of Mild TBI - so we get a better look at "dosage" of TBI.

Thank you to reviewer 2 for comments. No changes were required.
