# Supplementary material for: Associations between significant head injury in male juveniles in prison in Scotland UK and cognitive function, disability and crime: A cross sectional study
Source: PLoS One. 2023 Jul 12;18(7):e0287312. doi: 10.1371/journal.pone.0287312 (PMC10337871; doi:10.1371/journal.pone.0287312)
Supplement: Attachment — Submitted filename: Response to Reviewers_Apr23docx.docx [file pone.0287312.s006.docx]

**Response to Reviewers Comments April 2023**

**PONE-D-22-23246**
Associations between Significant Head Injury  in Male Juveniles in Prison in Scotland UK and Cognitive Function, Disability and Crime: A Cross sectional study

**Editors Comments**
No additional changes requested

**Reviewer #1**: This reviewer was happy with revisions made at the last submission. No further changes required.

**Reviewer #2:** This is a new reviewer -who was enthusiastic about the paper and requested two minor changes.

1.I appreciate the brief introduction to the paper and I think the authors hit all the necessary key points, but I do think that they do not provide enough detail surrounding previous studies examining not only the association between TBI and cognitive function/behavior problems, but also the underlying mechanisms that underlie such associations. This list is not exhaustive (and I’m not asking the authors to perform a comprehensive literature review by any means) but here are a few studies that may be relevant and can be collectively summarized for interested readers who may want to dig deeper into this topic:

Sariaslan, A., Lichtenstein, P., Larsson, H., & Fazel, S. (2016). Triggers for violent criminality in patients with psychotic disorders. JAMA Psychiatry, 73(8), 796–803. <https://doi.org/10.1001/jamapsychiatry.2016.1349>

Sariaslan, A., Sharp, D. J., D’Onofrio, B. M., Larsson, H., & Fazel, S. (2016). Long-Term Outcomes Associated with Traumatic Brain Injury in Childhood and Adolescence: A Nationwide Swedish Cohort Study of a Wide Range of Medical and Social Outcomes. PLoS Medicine, 13(8), 15–19. <https://doi.org/10.1371/journal.pmed.1002103>

Schwartz, J. A. (2021). A Longitudinal Assessment of Head Injuries as a Source of Acquired Neuropsychological Deficits and the Implications for Criminal Persistence. Justice Quarterly, 38(2), 196–223. <https://doi.org/10.1080/07418825.2019.1599044>

Schwartz, J. A., Connolly, E. J., & Brauer, J. R. (2017). Head Injuries and Changes in Delinquency from Adolescence to Emerging Adulthood: The Importance of Self-control as a Mediating Influence. Journal of Research in Crime and Delinquency, 54(6), 869–901. <https://doi.org/10.1177/0022427817710287>

Schwartz, J. A., Connolly, E. J., & Valgardson, B. A. (2018). An evaluation of the directional relationship between head injuries and subsequent changes in impulse control and delinquency in a sample of previously adjudicated males. Journal of Criminal Justice, 56(August), 70–80. <https://doi.org/10.1016/j.jcrimjus.2017.08.004>

Schwartz, J. A., Wright, E. M., Spohn, R., Campagna, M. F., Steiner, B., & Epinger, E. (2022). Changes in Jail Admissions Before and After Traumatic Brain Injury. Journal of Quantitative Criminology, 38, 1033–1056. <https://doi.org/10.1007/s10940-021-09524-7>

Just to be clear, I’m not suggesting the authors should dramatically expand their literature review, but just a couple of additional sentences aimed at summarizing the existing research in this area would be beneficial.

We have added a couple of sentences in the Introduction in line with the reviewer’s suggestion. We had also cited reviews here for the interested reader, which are more wide ranging (eg Williams et al 2018) and allow the reader easy entry to dig into further reading but have also added some further references as suggested.

2.Can the authors provide any additional information pertaining to how similar the demographc characteristics of their sample are compared to the overall population of juveniles at the correctional facility examined?

We agree with the reviewer that this is an important issue and worth providing more detail. In the Methods section on page 5 we indicated that 98% of juvenile males in prison in Scotland are resident in HMYOI Polmont and are therefore likely to be representative of this population in Scotland. We have added more information comparing demographic data from Scottish Government statistics on juvenile males in prison on page 10 and in table 1 and have added a more detailed table on page 27 of the supplement with links to the relevant government database.

**Reviewer #3:**  No changes requested.
